# Mixed reality for teaching catheter placement to medical students: a randomized single-blinded, prospective trial

**DOI:** 10.1186/s12909-020-02450-5

**Published:** 2020-12-16

**Authors:** D. S. Schoeb, J. Schwarz, S. Hein, D. Schlager, P. F. Pohlmann, A. Frankenschmidt, C. Gratzke, A. Miernik

**Affiliations:** Department of Urology, Faculty of Medicine, Medical Center – University of Freiburg, Hugstetter Str. 55, 79106 Freiburg, Germany

**Keywords:** Education, medical, Simulation training, Urinary catheters, Urologic surgical procedures

## Abstract

**Background:**

Cost-effective methods to facilitate practical medical education are in high demand and the “mixed-reality” (MR) technology seems suitable to provide students with instructions when learning a new practical task. To evaluate a step-by-step mixed reality (MR) guidance system for instructing a practical medical procedure, we conducted a randomized, single-blinded prospective trial on medical students learning bladder catheter placement.

**Methods:**

We enrolled 164 medical students. Students were randomized into 2 groups and received instructions on how to perform bladder catheter placement on a male catheterization training model. One group (107 students) were given their instructions by an instructor, while the other group (57 students) were instructed via an MR guidance system using a Microsoft HoloLens. Both groups did hands on training. A standardized questionnaire covering previous knowledge, interest in modern technologies and a self-evaluation was filled out. In addition, students were asked to evaluate the system’s usability. We assessed both groups’s learning outcome via a standardized OSCE (objective structured clinical examination).

**Results:**

Our evaluation of the learning outcome revealed an average point value of 19.96 ± 2,42 for the control group and 21.49 ± 2.27 for the MR group - the MR group’s result was significantly better (*p* = 0.00). The self-evaluations revealed no difference between groups, however, the control group gave higher ratings when evaluating the quality of instructions. The MR system’s assessment showed less usability, with a cumulative SUS (system usability scale) score of 56.6 (lower half) as well as a cumulative score of 24.2 ± 7.3 (*n* = 52) out of 100 in the NASA task load index.

**Conclusions:**

MR is a promising tool for instructing practical skills, and has the potential to enable superior learning outcomes. Advances in MR technology are necessary to improve the usability of current systems.

**Trial registration:**

German Clinical Trial Register ID: DRKS00013186

## Background

Technological progress has changed the medical field dramatically in recent decades, transforming the training of practical medical and surgical tasks. There is now a variety of training models [1–3] available. However, training often still takes place on the patient [4] and simulators and well-equipped skills labs are not widely available, as they are costly and require additional resources. While most university hospitals in Germany are equipped with skills labs for their students, simulation-based training for residents to perform more specialized and demanding medical tasks, especially at non-university hospitals, remains very limited. Therefore, cost-effective methods to facilitate practical medical education – methods that can potentially reduce the number of human instructors necessary - are in high demand. “Mixed reality” (MR) is a new technology that expands our perception of our surroundings through virtual objects or information that are projected into the user’s field of vision. The term refers to a continuum encompassing Virtual Reality (VR) and Augmented Reality (AR). There is strong evidence of the practicality of MR technology in a clinical setting, and numerous successful trials have applied this technology [5–7]. The information is usually delivered via a portable head-mounted display that can be activated nearly everywhere. For the training of practical skills, an MR system seems most suitable to provide students with instructions when learning skills on a training model or performing a practical task for the first time in a clinical setting. While studies have reported that simulation-based medical education can be more effective than traditional clinical education [8], the number of studies addressing this issue and making a “one to one” comparison is still small, since implementing simulation training often requires many implementation steps and necessitates that the technology accommodate the local technology standards at hand [9] There have been no randomized studies benefiting from the application of MR technology for practical medical education til now, and while it is generally agreed that MR has strong potential as a useful teaching tool for medical trainees, the scientific evidence is lacking. Our trial aimed to deliver an objective and subjective evaluation of learning outcomes applying an MR system to train medical students to perform bladder catheter placement. In addition, we designed the trial to also assess the usability of the MR system and students’ learning experience.

## Methods

### Participants

Between August 2017 and August 2018 we recruited 164 medical students who were doing their rotation in urology in our Department of Urology at the University of Freiburg Medical Center. Students were enrolled between their 4 and 5th year in a 6-year M.D. (medical doctor) program. Study participation was open to all medical students qualified to participate in the urological rotation; there were no exclusion criteria. While participating in the urological rotation is mandatory for medical students who have passed the theoretical exam in urology after attending a lecture series, neither inclusion nor performing in this study was associated with their regular curriculum. All participants consented to participate prior to their inclusion in the study.

### Study design

This study was designed adhering to the CONSORT guidelines, approved by our local ethics committee, and conducted in accordance with the ethical standards laid down in the 1964 Declaration of Helsinki and its later amendments. It was registered prospectively in the German Clinical Trial Register (ID:DRKS00013186). Sample size and the ~ 2:1 ratio were calculated after concluding a pilot study using a two sided t-test (significance level *p* ≤ 0.05). Students were randomized into 2 study groups (numbers lottery) and given 30 min of instruction on how to perform bladder catheter placement. The instructions given to both groups were identical and standardized (supplementary file 5), and followed the recommendations on bladder catheter placement [10]. Bladder catheter placement was carried out using a male catheterization-training model (male catheter model type LM29, Erler-Zimmer GmbH & Co. KG, Lauf, Germany) and a Tiemann catheter CH16 (UROMED Kurt Drews KG, Oststeinbek, Germany). The control group was given instructions by an instructor, while the other received instructions through a video-based AR guidance system. Both groups engaged in hands-on training during those 30 min. The MR group performed bladder catheter placement while receiving instructions from the MR system. The control group got their instructions from a human instructor and then performed the catheter placement under the instructor’s supervision. All the nursing instructors participating in this study were fully trained staff nurses in the urology department with 10 years of experience in teaching this class.

### Mixed reality guidance system

**Fig. 1:**
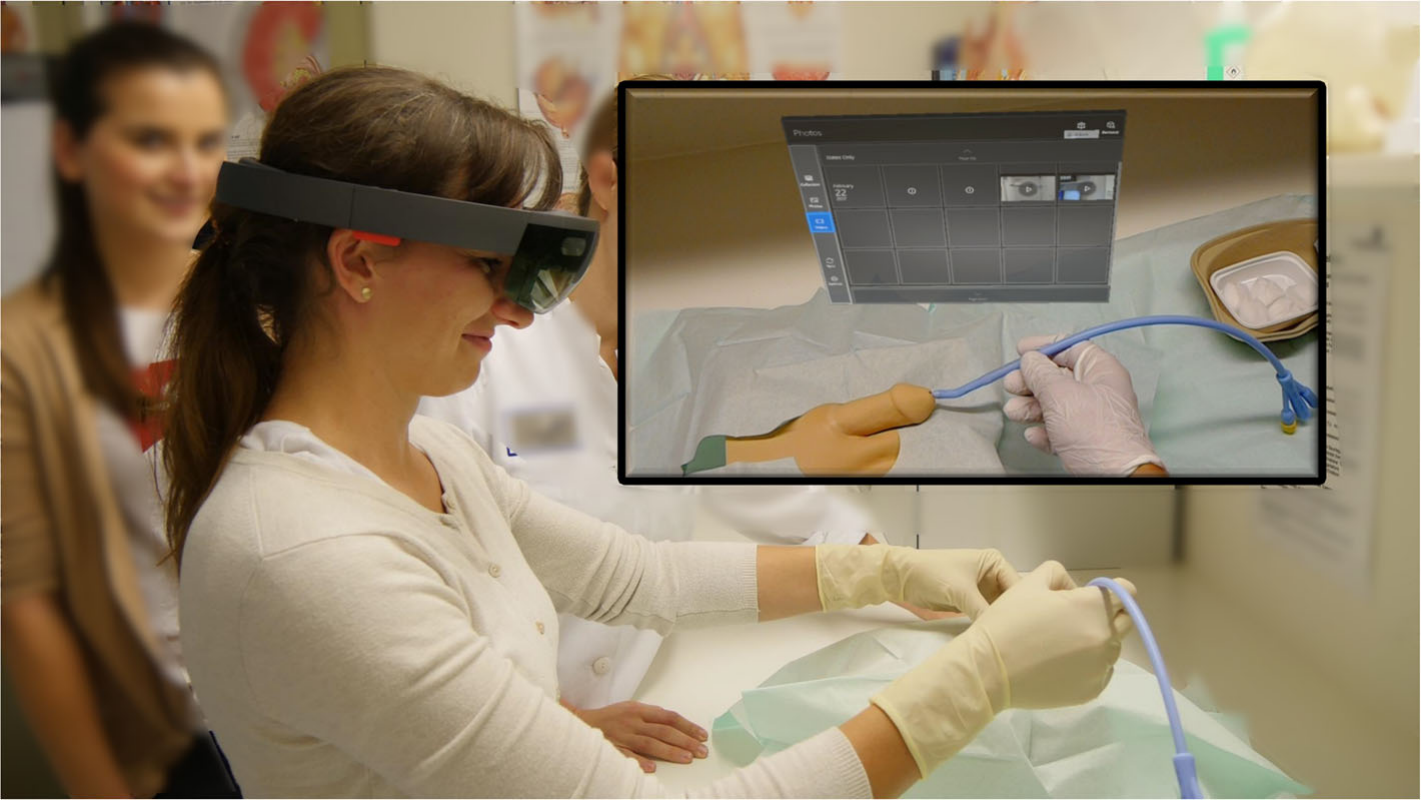
Application of the Mixed Reality (MR) system in our study. Within the picture the view through the Microsoft HoloLens is depicted with the menu of the MR system for video selection opened. (Copyright of the picture by the first author)

Students randomized to the MR group received instructions exclusively through an MR system. Instructions were displayed through a head-mounted display (HMD) using the Microsoft Hololens (Microsoft Corporation, Redmond, USA). After an introduction (indications, how to best prepare the patient, appropriate setting), the system provided step-by-step instructions on how to prepare the materials in a sterile manner, followed by guidance through the placement process. To accommodate different performance speeds, instructions were provided on demand, and the system was programmed to stop automatically until further instructions were requested. All instructions were provided through combined video-based visual and audio guidance (Fig. 1). No student received any additional support when performing the task, however, technical support in applying the MR system was available from us study authors. This pedagogical concept was developed under the guidance of a certified expert with a master’s degree in medical education (A. F.) and relies on current recommendations for bladder catheter placement [10].

### Evaluation

We conducted a standardized, non-timed OSCE (Objective structured clinical examination) after 3 days (primary outcome measure) to assess learning outcomes. The structure of this OSCE was derived from a previously evaluated, published format [11]. As the instructor giving the OSCE was unaware about how any participant had been instructed, this was a single-blinded setting. This OSCE was not part of the urological rotation curriculum, and it was only implemented to evaluate learning outcomes related to this study.

A total score of 24 points was possible. The OSCE was drafted independently from the MR system to prevent confounding (supplementary file 4). Students also filled out a standard questionnaire (supplementary file 1) before and after the instructions. Recorded demographic data included gender, age and semester in medical school, as well as intended residency and any experience as a paramedic or nurse. For the self-evaluations (secondary outcome measure), students ranked their ability in the given task; this covered their teaching preference and an evaluation of their learning experience during the study (a 6-point Likert scale was used to avoid a neutral position). Furthermore, students using the MR system were asked to evaluate the system via the NASA Task load index [12] and the standard system-usability scale (SUS) [13] in the German language (supplementary files 2 and 3). For the SUS, students had to answer 10 questions regarding complexity, usability, functional integrity, consistency and applicability to everyday teaching on a scale from 1 to 5. The SUS’ total cumulative score was calculated according to Brooke et al. [14]. All data was anonymized; there were no names of participants recorded.

### Statistical evaluation

Statistical analyses were carried out using the Statistical Package for Social Sciences software (SPSS®, Version 20.0, Chicago, IL, USA). Differences between groups were analyzed by Kruskal-Wallis test for non-parametric data and in case of significant differences confirmed by Mann-Whitney test. Numeric data differences were analyzed via ANOVA and in case of significance, confirmed by T-Test. *P*-values < 0.05 were considered to be significant. All data are represented as mean ± standard deviation.

## Results

### Student cohort

Of the 164 students enrolled in our study, 95 were female and 69 male. Fifty-nine students were randomized to the MR group and 105 students were allocated to the control group. Prior experience with bladder catheter placement on a model was claimed by 84.1% of participants. Study semesters ranged from 8 to 12 with a majority of students (> 50%) having studied more than 4 years. Average age was 25.2 ± 2.8 with a range from 21 to 39 years. There was no significant difference in gender distribution (*p* = 0.7), age distribution (*p* = 0.15) or previous medical experience as a nurse or a paramedic (*p* = 0.57) between the MR group and controls. Prior bladder catheter-placement experience on a model (*p* = 0.50) or on a patient (*p* = 0.33), did not differ either. Nor were there any differences in the affinity with new technologies (possession of smartphone (*p* = 0.45), possession of a gaming console (*p* = 0.76), possession of a tablet (*p* = 0.45)) or previous experience with AR devices (*p* = 0.55) and IT-based technology (knowledge of a programming language (*p* = 0.44), usage of online learning platforms (*p* = 0.18) and social media (*p* = 0.93)). All results are depicted in Table 1.

**Table 1:** Demographics of student cohort and comparison between study and control group

	Control group	Study group	***P*** value
**Students (n=)**	105	59	n. a.
**Gender (male/female in %)**	41/59	44/56	0.7
**Age (mean ± standard deviation)**	25.49 ± 3.08	24.78 ± 2.24	0.15
**Semester in medical school (mean ± standard deviation)**	8.88 ± 1.18	9.1 ± 1.2	0.26
**Nursing/paramedic experience (in%)**	11.4	13.6	0.57
**Possesses a smartphone (in%)**	99	100	0.45
**Possesses a tablet (in%)**	61.9	67.8	0.45
**Possesses a gaming console (in%)**	23.8	27.1	0.76
**Active on social media (in%)**	81.9	81.4	0.93
**Knowledge of programming language (in%)**	15.2	11.9	0.44
**Previous contact with AR/VR (in%)**	15.2	11.9	0.55

### Teaching preference and learning experience

All students filled out a standard questionnaire prior to and after the teaching session. When ranking teaching methods, training directly on a patient was given an average of 2.57 ± 1.3 points, while using a training model was given 2.92 ± 1.3. When asked to rank an instructor’s instructions versus those through digital media, 85.9% of students leaned towards the instructor (average of 2.24 ± 1.2). No significant difference appeared in student preference prior to or after the study, except for a slightly higher preference towards learning on the patient (*p* = 0.036) in the MR group prior to our study that disappeared afterwards. Results are depicted in Table 3.

Evaluation after the training course showed that both groups found the training concept good with a score > 3 in 84.7% in the MR group and 92.6% for the control group (*p* = 0.181). We identified significant differences in how participants rated confusing instructions (*p* = 0.002), fulfillment of expectations (*p* = 0.003) and level of enjoyment (*p* = 0.004), with the control group delivering better evaluations. However, with average point values of 2.53 ± 1.4, (MR group; 77.9% enjoyed or greatly enjoyed the class) vs. 1.9 ± 1.1 (control group; 93.3% enjoyed or greatly enjoyed the class) for class enjoyment, 2.9 ± 1.4 (MR group; 67.8% fulfilled expectations) vs. 2.25 ± 1.2 (control group; 87.6% fulfilled expectations) for fulfilled expectations and 4.5 ± 1.6 (MR group; 72.9% reported no confusing statements) vs. 5.1 ± 1.4 (control group, 81,2% reported no confusing statements) for receiving confusing instructions, students in both groups gave their learning experience a generally positive assessment. All results are depicted in Table 2.

**Table 2:** Results of teaching evaluation in study and control group

	Control group	Study group	***P*** value*
**Training/exercise time was sufficient:**	3.0 ± 1.5	3.2 ± 1.6	0.646
**The training encouraged me to perform on a patient**	2.1 ± 1.2	2.4 ± 1.2	0.098
**The training/exercise was fun**	1.9 ± 1.2	2.5 ± 1.4	**0.004**
**The teacher held a monologue**	5.0 ± 1.3	4.7 ± 1.7	0.420
**A clear concept was perceptible during the training session**	2.2 ± 1.2	2.4 ± 1.2	0.129
**The teacher motivated me to actively participate:**	1.73 ± 1.1	2.3 ± 1.2	**0.004**
**My expectations were fulfilled:**	2.3 ± 1.2	2.9 ± 1.4	**0.003**
**The content was confusing:**	5.1 ± 1.4	4.5 ± 1.6	**0.002**

*Significant differences are marked in bold font. Values correspond to a 6 point Likert scale (1 = “strongly agree” to 6 = “strongly disagree with that statement)

### Self-evaluation

The mean value before the training session was 2.43 ± 1.4 concerning bladder catheter placement on a patient. After training, this value increases significantly (*p* < 0.001) to a value of 3.4 ± 1.3, although there was no significant difference between groups. Similarly, students ranked their ability to perform bladder catheter placement on a training model (3.16 ± 1.3 before vs. 4.47 ± 1.3 after; *p* < 0.001), their theoretical knowledge of bladder catheter placement (3.59 ± 1.3 before vs. 4.77 ± 1.1 after; *p* = 0.00) and their ability to do the procedure in a sterile fashion (3.61 ± 1.3 before vs. 4.47 ± 1.0 after; *p* < 0.001). Our group comparison revealed that the MR group felt more confident performing bladder catheter placement independently before our study’s training session (*p* < 0.001). However, confidence levels after the training were equal in both groups. Nevertheless, the control group reported slightly more confidence in their theoretical knowledge of bladder catheter placement (2.12 ± 1.1) than the MR group (2.44 ± 1.0; *p* = 0.01) after the training, while those values before training did not differ (*p* = 0.58). We detected no significant difference in self-evaluations before and after the training in any of the other parameters nor in their change during the study. Detailed results are depicted in Table 3.

**Table 3:** Results of self-evaluation in study and control group before and after the training

	Control group		Study group		***P*** value*	
	Before	After	Before	After	Before	After
**Preference for training on dummy**	2.90 ± 1.3	2.74 ± 1.2	2.95 ± 1.3	2.93 ± 1.4	0.75	0.38
**Preference for training on patient**	2.72 ± 1.3	2.74 ± 1.2	2.31 ± 1.2	2.68 ± 1.3	0.04	0.87
**Preference for instructor based training**	2.24 ± 1.3	2.41 ± 1.4	2.24 ± 1.3	2.24 ± 1.2	0.76	0.53
**Residency goal: urology (%)**	2.9	2.4	1.7	1.7	0.50	0.67
**Residency goal: Surgery residency (%)**	11.0	10.5	11.9	11.9	0.78	0.68
**Able to perform on a dummy**	2.52 ± 1.2	3.85 ± 1.4	2.63 ± 1.0	3.95 ± 1.3	0.69	0.35
**Able to perform on a patient**	3.5 ± 1.3	4.56 ± 1.5	3.83 ± 1.2	4.68 ± 1.3	0.80	0.10
**Theoretical skill-level**	2.12 ± 1.1	3.4 ± 1.4	2.44 ± 1.0	3.51 ± 1.2	0.58	**0.01**
**Able to perform while maintaining sterility**	2.52 ± 1.2	3.45 ± 1.4	2.59 ± 0.9	3.36 ± 1.1	0.62	0.41
**Able to perform independently**	3.51 ± 1.5	5.93 ± 0.4	3.69 ± 1.2	4.53 ± 1.3	**0.00**	0.45
**future specialty already decided**	3.43 ± 1.8	3.5 ± 1.7	3.49 ± 1.9	3.56 ± 1.8	0.86	0.84

All values are given as mean ± standard deviation unless marked otherwise. Preferences in teaching were ranked on a Likert scale from 1 to 6 (1 = “fully agree” to 6 = “fully disagree” with the given statement, in this case the preference for a specific training method); ability and skill perception was ranked on a Likert scale from 1 to 6 (1 = “not good at all” to 6 = “very good”)

* Significant differences are marked in bold font

### OSCE results

Our OCSE evaluation revealed an average 19.96 ± 2,42 points for the control and 21.49 ± 2.27 for the MR group - a significantly better score for the latter (*p* = 0.00). Cumulative learning outcome was high in both groups, with an average 20.51 ± 2.57 out of 24 points. Comparison of the performance between students with previous experience in paramedics (*p* = 0.442) or nursing (*p* = 0.888) and those with no prior medical education revealed no difference Moreover, we detected no difference within the MR group’s students with prior AR/VR technology experience (*p* = 0.115), ownership of a gaming console (*p* = 0.200), regular occupation with computer games (*p* = 0.232), or knowledge of a programming language (*p* = 0.224).

### Evaluation of the MR system

Immediately after completing MR training, all students randomized to the MR group were asked to evaluate the proposed MR technology. In this context, 40.2% of participants reported finding the system difficult to use, and 28.9% believed they could only use the system with an instructor’s help, a finding reflecting poor applicability for everyday teaching. However, only 10.2% believed they would need extensive instructions. The total cumulative SUS score was 56.6 (*n* = 52). SUS results are depicted in Table 4. Analysis of the NASA Task load index answers estimating how strenuous it is to use the MR system revealed a cumulative score of 24.2 ± 7.3 (*n* = 52) out of a maximum of 100 (=highest perceived workload). While mental (3.58 ± 1.8) and physical demand (2.48 ± 1.9) as well as time demand (4.09 ± 1.8), perceived success (4.30 ± 2.26) and overall effort (4.37 ± 2.0) all attained an average below 5, students ranked their emotional stress (frustration, anger) higher with 5.39 ± 2.8. We found no significant difference among students with previous AR/VR technology experience (*n* = 7), programming capabilities (*n* = 7), ownership of a gaming console (*n* = 16) or regular occupation with computer games (*n* = 11) for any of the categories of our evaluation tools (SUS, NASA Task load index).

**Table 4:** Evaluation of the applied Mixed Reality (MR) system using the standardized system usability scale (SUS)

	Strongly disagree	Disagree	Neutral	Agree	Strongly agree	Average point value awarded
**I’d like to use this system frequently**	16,9%	25,4%	30,5%	20,3%	6,8%	2.9/5
**I found the system unnecessarily complex.**	15,3%	37,3%	20,3%	20,3%	6,8%	2.6/5
**I found the system easy to use.**	11,9%	27,1%	28,8%	25,4%	6,8%	3.1/5
**I think I’d need support from a technician to be able to use this system**	23,7%	27,1%	20,3%	13,6%	15,3%	2.7/5
**I found the various functions in this system well integrated**	8,5%	23,7%	35,6%	25,4%	6,8%	3/5
**I thought this system was full of inconsistencies..**	15,3%	33,9%	27,1%	18,6%	5,1%	2.6/5
**I imagine most people could learn to use this system very quickly.**	8,5%	11,9%	32,2%	25,4%	22,0%	3.5/5
**I found the system very cumbersome to use.**	11,9%	32,2%	22,0%	27,1%	6,8%	2.9/5
**I felt very confident using the system.**	10,2%	23,7%	37,3%	20,3%	8,5%	3.1/5
**I had to learn a lot of before I could begin to use this system.**	33,9%	35,6%	20,3%	6,8%	3,4%	2.1/5

## Discussion

In this randomized, prospective, single-blinded study, we applied and evaluated an interactive, video-based MR system. To our knowledge, we are the first to have conducted a prospective, randomized study in the field of urology investigating MR’s value for teaching practical medical skills. We assessed its efficiency through self-evaluation as well as via an OSCE to enable both subjective and objective means of measuring learning outcome. In addition, students were asked to evaluate the system’s usability and their learning experience. Our results reveal similar outcomes in the self-evaluation and a slightly better learning outcome in the MR group in the OSCE exam. Students’ learning-experience evaluations were also similar between the MR and control groups, receiving a generally positive appraisal. In addition, the MR system’s usability, as evaluated through SUS and NASA Task load index revealed a mid range result for both scores. While several pilot studies have applied this technology as a supporting tool for various surgical applications [15] including liver surgery [16], dentistry [17], tele-surgery [18] and robotic surgery [19], randomized studies investigating this technology’s impact are rare. In this context, MR technology has been applied for teaching anatomical structures, and a prospective study demonstrated the non-inferiority of this technology compared to a tablet-based approach [20]. The field of laparoscopic surgery frequently applies MR technologies in simulation training to teach practical skills [21], and various simulators are commercially available. A recent study also using the Microsoft Hololens demonstrated improved outcome parameters when used as a visual aid during ureteroscopy [22]. An advantage of this technology is its functionality without requiring teaching personnel, making it an efficient means of training independent of the fixed time schedules normally required in a traditional lecture-instructional context. In our study, the AR system provided all the information needed to carry out the task at hand, as well as its theoretical basis. Technical supporting staff was, however, provided, since most students needed initial help utilizing the HMD (Microsoft HoloLens). Once the students were instructed, the system functioned autonomously. To apply this technology in a broader sense, a student can receive initial instructions and then learn a variety of practical skills without needing an instructor, making teaching more flexible and cost-efficient. However, the current shortcomings of the hardware (uncomfortable, heavy HMDs, short battery life, complicated handling) as well as the necessary infrastructure (fast, reliable internet connection, EDV specialists, etc.) still make it difficult to employ MR widely in everyday teaching. In our study, participants often needed help in handling the device during the procedures, and technical problems did occur. While these difficulties were easy to overcome due to the attending technical personnel, it might cause a problem for students learning autonomously. This fact is represented in the overall SUS score of ~ 57, which makes the usability of our system only marginally acceptable [23]. We also believe that the good (but significantly lower) evaluation of the MR teaching experience can be attributed to our system’s usability. This is supported by the identical student self-evaluations. A further advantage of a standardized teaching approach using an MR system is its consistency. While an instructor might vary in emphasizing different aspects or alter the content, a standardized MR teaching tool guarantees the same content for every application. This point is reflected in the significantly better OSCE exam result. In our study, lower ratings were entirely caused by 3 different points on the OSCE checklist, namely that our instructor repeatedly failed to provide this information, or failed to emphasize those aspects. In summary, our results show, that the MR system provides a comparable learning outcome to conventional training, with a higher degree of consistency. However, our study has specific limitations. While providing an interactive MR system, we used a video-based approach without the capability of recognizing objects, hence it does not truly “augment” the environment by providing a system that actually interacts with real objects (=Augmented Reality). The technology applied in our study is therefore categorized as a “Mixed Reality” system [15] We believe, however, that stronger interaction with the environment will improve teaching outomes and support the our study’s message, namely that this technology is a practical solution for teaching medical procedures, and the hardware currently available enables an acceptable degree of usability. While continuous development is necessary, resources need to be allocated to implement this technology further and spur on technological progress. We did not include a control group employing an alternative technological solution to an HMD to display information (eg, tablet computer). Such solutions may also be feasible and yield acceptable learning outcomes. However, we know of no other technologies that have as much potential as HMDs for advancing the development of interactive teaching systems, with the capacity of both instruments enabling on-demand supervisor feedback and the direct implementation of automatic-assessment tools through object recognition. We also conducted no long-term follow up, since study participants we assured that a poor performance would not affect their grades in their urology rotation. As we therefore decided to use anonymized data, no long-term follow up was possible. Also, we are obliged to mention that our statistical calculations were performed without a method for *p*-value correction, which may have affected our results evaluating the learning experience. Since our data reveals a generally positive evaluation from both groups, it is safe to assume that the learning experience the MR system provided was evaluated by the MR group at least as positively as the control group had done.

## Conclusions

MR is a efficient tool for instructing bladder catheter placement. It is a promising technology for teaching practical medical tasks in general, as it delivers learning outcomes resembling those of a human instructor. Still more developmental progress with MR technology is needed to improve the usability of current MR systems.

## Supplementary Information

**Additional file 1.** Standardized questionnaire applied in this study in German language (original version) and English translation.

**Additional file 2.** NASA Task Load Index questionnaire applied in this study in German language (original version) and English version.

**Additional file 3.** System usability scale questionnaire applied in this study in German language (original version) and English version.

**Additional file 4.** OSCE checklist applied in this study in German language (original version) and English version.

**Additional file 5.** General steps for bladder catheter placement as thaught in this study.

## Data Availability

The datasets generated and/or analyzed during the current study are not publicly available due to data protection laws but are available from the corresponding author on reasonable request.

## Abbreviations

AR: Augmented Reality
HMD: Head Mounted Display
MR: Mixed reality
NASA: National Aerospace Agency
OSCE: Objective structured clinical examination
SPSS: Statistical Package for Social Sciences software
SUS: System usability scale
VR: Virtual Reality
