# Supplementary material for: Mixed reality for teaching catheter placement to medical students: a randomized single-blinded, prospective trial
Source: BMC Med Educ. 2020 Dec 16;20:510. doi: 10.1186/s12909-020-02450-5 (PMC7745503; doi:10.1186/s12909-020-02450-5)
Supplement: Supplementary file 1 — Additional file 1. Standardized questionnaire applied in this study in German language (original version) and English translation. [file 12909_2020_2450_MOESM1_ESM.docx]

**Studie „Evaluation von Augmented Reality (AR) als Lehrmedium zur Vermittlung praktischer Fertigkeiten anhand der Dauerkatheter Anlage im Rahmen des urologischen Blockpraktikums“**

NR:

**Fragebogen zur Selbstevaluation vor Praktikumsbeginn**

**1. Persönliche Daten/Allgemeine Fragen:**

Alter:__________ Geschlecht:____________ Semesterzahl:______________

Frühere Tätigkeit im Pflegedienst: □ja □nein

Frühere Tätigkeit im Rettungsdienst: □ja □nein

Praktische Fähigkeiten erlerne ich sehr leicht: stimmt völlig □ □ □ □ □ □ stimmt nicht

Praktische Fähigkeiten erlerne ich lieber am Model: stimmt völlig □ □ □ □ □ □ stimmt nicht

Praktische Fähigkeiten erlerne ich lieber am Patient: stimmt völlig □ □ □ □ □ □ stimmt nicht

Ich ziehe den persönlichen Unterricht digitalen Medien vor: stimmt völlig □ □ □ □ □ □ stimmt nicht

Ich strebe eine urologische Facharztausbildung an: □ja □nein

Ich strebe eine chirurgische Facharztausbildung an: □ja □nein

Mein Facharztziel steht bereits fest: stimmt völlig □ □ □ □ □ □ stimmt nicht

Mein Facharztziel ist: ____________________________________

**2. Affinität zu „Neuen Medien“**

Ich besitze ein Smartphone: □ja □nein

Ich besitze ein Tablet: □ja □nein

Ich bin suche Informationen vor allem über:

□Fernsehen □Radio □Internet □Bücher □Apps □Zeitungen

Ich nutze das Internet zur Lernzwecken: □ja □nein

Ich lerne vor allem mittels digitaler Medien: stimmt völlig □ □ □ □ □ □ stimmt nicht

Ich spiele regelmäßig Computerspiele: □ja □nein

Ich besitze eine Computerspielkonsole: □ja □nein

Ich beherrsche eine Programmiersprache □ja □nein

Ich hatte bereits Kontakt mit AR/VR Anwendungen: □ja □nein

NR:

Ich bin auf „sozialen Medien“ aktiv: □ja □nein

**3. Tätigkeitsspezifische Fragen**

Wie oft hast du bereits eine BDK Anlage am Model durchgeführt:_____________________________

Wie oft hast du bereits eine BDK Anlage am Patient durchgeführt: ____________________________

Wie sicher fühlst du dich bei der BDK Anlage am Model? völlig unsicher □ □ □ □ □ □ völlig sicher

Wie sicher kennst du den theoretischen Ablauf? völlig unsicher □ □ □ □ □ □ völlig sicher

Wie sicher kannst du die Sterilität bei erhalten? völlig unsicher □ □ □ □ □ □ völlig sicher

Wie sicher fühlst du dich bei der BDK Anlage am Patient? völlig unsicher □ □ □ □ □ □ völlig sicher

Ich kann selbstständig eine BDK Anlage durchführen: stimmt völlig □ □ □ □ □ □ stimmt nicht

**Fragebogen zur Selbstevaluation NACH erfolgtem Praktikum**

NR:

**1. Tätigkeitsspezifische Fragen**

Wie sicher fühlst du dich bei der BDK Anlage am Model? völlig unsicher □ □ □ □ □ □ völlig sicher

Wie sicher kennst du den theoretischen Ablauf? völlig unsicher □ □ □ □ □ □ völlig sicher

Wie sicher kannst du die Sterilität bei erhalten? völlig unsicher □ □ □ □ □ □ völlig sicher

Wie sicher fühlst du dich bei der BDK Anlage am Patient? völlig unsicher □ □ □ □ □ □ völlig sicher

Ich kann selbstständig eine BDK Anlage durchführen: stimmt völlig □ □ □ □ □ □ stimmt nicht

**2. Kursspezifische Fragen (NUR BDK Anlage, nicht ganzen Kurs bewerten)**

Praktische Fähigkeiten erlerne ich lieber am Model: stimmt völlig □ □ □ □ □ □ stimmt nicht

Praktische Fähigkeiten erlerne ich lieber am Patient: stimmt völlig □ □ □ □ □ □ stimmt nicht

Ich ziehe den persönlichen Unterricht digitalen Medien vor: stimmt völlig □ □ □ □ □ □ stimmt nicht

Ich strebe eine urologische Facharztausbildung an: □ja □nein

Ich strebe eine chirurgische Facharztausbildung an: □ja □nein

Mein Facharztziel steht bereits fest: stimmt völlig □ □ □ □ □ □ stimmt nicht

Die Übungs-/Unterrichtszeit war ausreichend: stimmt völlig □ □ □ □ □ □ stimmt nicht

Die Demo/Übung der BDK Anlage hat Spaß gemacht: stimmt völlig □ □ □ □ □ □ stimmt nicht

Der Kurs ermutigte mich die BDK Anlage am Patient
durchzuführen: stimmt völlig □ □ □ □ □ □ stimmt nicht

Der Dozent hat Monologe gehalten: stimmt völlig □ □ □ □ □ □ stimmt nicht

Bei der Vermittlung praktischer Fähigkeiten war ein
einheitliches Konzept erkennbar: stimmt völlig □ □ □ □ □ □ stimmt nicht

Der Dozent hat aktiv zur Teilnahme motiviert: stimmt völlig □ □ □ □ □ □ stimmt nicht

Meine Erwartungen haben sich erfüllt: stimmt völlig □ □ □ □ □ □ stimmt nicht

Die Darstellung der Lerninhalte war unverständlich: stimmt völlig □ □ □ □ □ □ stimmt nicht

**Study „Evaluation of Augmeted Reality (AR) as teaching instrument for the mediation of practical skills using bladder catheter placement during urlogical clinical internship “**

NR:

***Questionnaire for self evaluation BEFORE training***

**1. Personal information/general questions**

Age:__________ Gender:____________ Semester:______________

Prior experience in nursing: □yes □no

Prior experience as paramedic: □yes □no

I learn practical skills easily: strongly agree □ □ □ □ □ □ strongly disagree

I prefer to learn practical skills on a dummy: strongly agree □ □ □ □ □ □ strongly disagree

I prefer to learn practical skills on a patient: strongly agree □ □ □ □ □ □ strongly disagree

I prefer being taught by an instructor via digital formats: strongly agree □ □ □ □ □ □ strongly disagree

I intend to start a urology residency: □yes □no

I intend to start a surgery residency: □yes □no

My future specialty has been decided: strongly agree □ □ □ □ □ □ strongly disagree

My specialty goal is: ____________________________________

**2. Affinity with „New Media Formats”**

I own a smartphone: □yes □no

I own a tablet: □yes □no

I look for information mostly through:

□TV □Radio □Internet □Books □Apps □Newspaper

I use the internet for studying: □yes □no

I use mostly digital formats for studying: strongly agree □ □ □ □ □ □ strongly disagree

I play video games regularly: □yes □no

I own a gaming console: □yes □no

I am able to apply a programming language: □yes □no

I had prior contact with AR/VR technology: □yes □no

NR:

I am active on “social media”: □yes □no

**3. Task-specific questions**

How often have you performed a bladder catheter placement on a dummy?:_____________________________

How often have you performed a bladder catheter placement on a patient?: ____________________________

How sure of yourself do you feel when performing on a dummy? very unsure □ □ □ □ □ □ very sure

How solid is your theoretical knowledge? not solid at all □ □ □ □ □ □ very solid

How confident are you in keeping sterile equipment sterile? not confident at all □ □ □ □ □ □ very confident

How sure of yourself do you feel when performing on a patient? very unsure □ □ □ □ □ □ very sure

I can put a bladder catheter in place without help: strongly agree □ □ □ □ □ □ strongly disagree

**Questionnaire for self evaluation AFTER training**

**1. Task-specific questions**

How sure of yourself are you when performing on a dummy? very unsure □ □ □ □ □ □ very sure

How solid is your knowledge of in theory? not solid at all □ □ □ □ □ □ very solide

How sure of yourself are you in keeping sterile equipment sterile? very unsure □ □ □ □ □ □ very sure

How secure are you when performing on a patient? very insecure □ □ □ □ □ □ very secure

I can put a bladder catheter in place without help: strongly agree □ □ □ □ □ □ strongly disagree

**2. Class-specific questions (only rate the bladder catheter-placement training)**

I prefer to learn practical skills on a dummy: strongly agree □ □ □ □ □ □ strongly disagree

I prefer to learn practical skills on a patient: strongly agree □ □ □ □ □ □ strongly disagree

I prefer being taught by an instructor via digital formats: strongly agree □ □ □ □ □ □ strongly disagree

I intend to start a urology residency: □yes □no

I intend to start a surgery residency: □yes □no

My future specialty has been decided: strongly agree □ □ □ □ □ □ strongly disagree

Training/practice time was sufficient: strongly agree □ □ □ □ □ □ strongly disagree

The training/practice was fun: strongly agree □ □ □ □ □ □ strongly disagree

The training encouraged me to perform on a patient: strongly agree □ □ □ □ □ □ strongly disagree

The teacher held a monologue: strongly agree □ □ □ □ □ □ strongly disagree

A clear concept was perceptible during the training session: strongly agree □ □ □ □ □ □ strongly disagree

The teacher motivated to actively participate: strongly agree □ □ □ □ □ □ strongly disagree

My expectations were fulfilled: strongly agree □ □ □ □ □ □ strongly disagree

The content was clearly understandable: strongly agree □ □ □ □ □ □ strongly disagree
