# Supplementary figures and images for: Mixed reality for teaching catheter placement to medical students: a randomized single-blinded, prospective trial
Source: BMC Med Educ. 2020 Dec 16;20:510. doi: 10.1186/s12909-020-02450-5 (PMC7745503; doi:10.1186/s12909-020-02450-5)

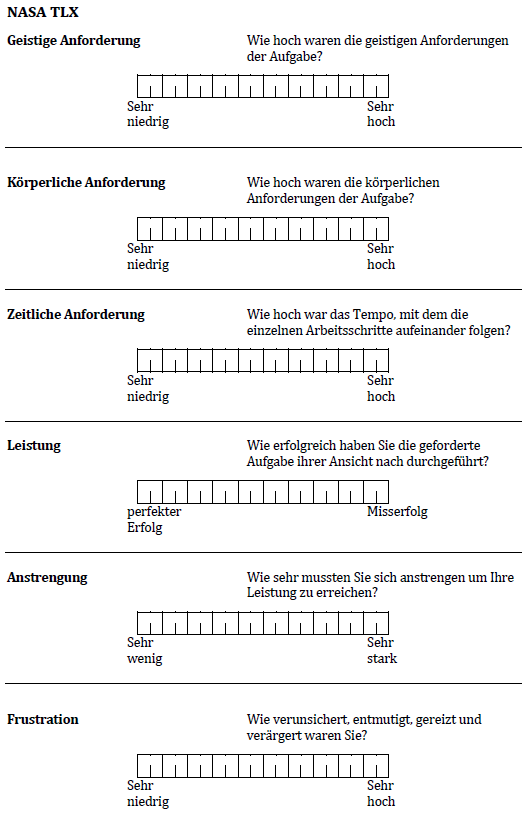

Supplement: Supplementary file 2 — Additional file 2. NASA Task Load Index questionnaire applied in this study in German language (original version) and English version. [file 12909_2020_2450_MOESM2_ESM.docx]
