# Supplementary material for: Mixed reality for teaching catheter placement to medical students: a randomized single-blinded, prospective trial
Source: BMC Med Educ. 2020 Dec 16;20:510. doi: 10.1186/s12909-020-02450-5 (PMC7745503; doi:10.1186/s12909-020-02450-5)
Supplement: Supplementary file 3 — Additional file 3. System usability scale questionnaire applied in this study in German language (original version) and English version. [file 12909_2020_2450_MOESM3_ESM.doc]

# Fragebogen zur System-Gebrauchstauglichkeit

1. Ich denke, dass ich das System gerne häufig benutzen würde.

| **Stimme  überhaupt nicht zu 1** | **2** | **3** | **4** | **Stimme voll zu  5** |
| --- | --- | --- | --- | --- |
|  |  |  |  |  |

1. Ich fand das System unnötig komplex.

| **Stimme  überhaupt nicht zu 1** | **2** | **3** | **4** | **Stimme voll zu  5** |
| --- | --- | --- | --- | --- |
|  |  |  |  |  |

1. Ich fand das System einfach zu benutzen.

| **Stimme  überhaupt nicht zu 1** | **2** | **3** | **4** | **Stimme voll zu  5** |
| --- | --- | --- | --- | --- |
|  |  |  |  |  |

1. Ich glaube, ich würde die Hilfe einer technisch versierten Person benötigen, um das System benutzen zu können.

| **Stimme  überhaupt nicht zu 1** | **2** | **3** | **4** | **Stimme voll zu  5** |
| --- | --- | --- | --- | --- |
|  |  |  |  |  |

1. Ich fand, die verschiedenen Funktionen in diesem System waren gut integriert.

| **Stimme  überhaupt nicht zu 1** | **2** | **3** | **4** | **Stimme voll zu  5** |
| --- | --- | --- | --- | --- |
|  |  |  |  |  |

1. Ich denke, das System enthielt zu viele Inkonsistenzen.

| **Stimme  überhaupt nicht zu 1** | **2** | **3** | **4** | **Stimme voll zu  5** |
| --- | --- | --- | --- | --- |
|  |  |  |  |  |

1. Ich kann mir vorstellen, dass die meisten Menschen den Umgang mit diesem System sehr schnell lernen.

| **Stimme  überhaupt nicht zu 1** | **2** | **3** | **4** | **Stimme voll zu  5** |
| --- | --- | --- | --- | --- |
|  |  |  |  |  |

1. Ich fand das System sehr umständlich zu nutzen.

| **Stimme  überhaupt nicht zu 1** | **2** | **3** | **4** | **Stimme voll zu  5** |
| --- | --- | --- | --- | --- |
|  |  |  |  |  |

1. Ich fühlte mich bei der Benutzung des Systems sehr sicher.

| **Stimme  überhaupt nicht zu 1** | **2** | **3** | **4** | **Stimme voll zu  5** |
| --- | --- | --- | --- | --- |
|  |  |  |  |  |

1. Ich musste eine Menge lernen, bevor ich anfangen konnte das System zu verwenden.

| **Stimme  überhaupt nicht zu 1** | **2** | **3** | **4** | **Stimme voll zu  5** |
| --- | --- | --- | --- | --- |
|  |  |  |  |  |

# System Usability Scale (SUS) questionnaire

1. I think that I would like to use this system frequently.

| **Strongly**  **disagree 1** | **2** | **3** | **4** | **Strongly**  **agree  5** |
| --- | --- | --- | --- | --- |
|  |  |  |  |  |

1. I found the system unnecessarily complex

| **Strongly**  **disagree**  **1** | **2** | **3** | **4** | **Strongly**  **agree  5** |
| --- | --- | --- | --- | --- |
|  |  |  |  |  |

1. I thought the system was easy to use

| **Strongly**  **disagree**  **1** | **2** | **3** | **4** | **Strongly**  **agree  5** |
| --- | --- | --- | --- | --- |
|  |  |  |  |  |

1. I think I would need the support of a technical person to be able to use this system

| **Strongly**  **disagree**  **1** | **2** | **3** | **4** | **Strongly**  **agree  5** |
| --- | --- | --- | --- | --- |
|  |  |  |  |  |

1. I found the various functions in this system were well integrated

| **Strongly**  **disagree**  **1** | **2** | **3** | **4** | **Strongly**  **agree  5** |
| --- | --- | --- | --- | --- |
|  |  |  |  |  |

1. I thought there was too much inconsistency in this system

| **Strongly**  **disagree**  **1** | **2** | **3** | **4** | **Strongly**  **agree  5** |
| --- | --- | --- | --- | --- |
|  |  |  |  |  |

1. I would imagine that most people would learn to use this system very quickly

| **Strongly**  **disagree**  **1** | **2** | **3** | **4** | **Strongly**  **agree  5** |
| --- | --- | --- | --- | --- |
|  |  |  |  |  |

1. I found the system very cumbersome to use

| **Strongly**  **disagree**  **1** | **2** | **3** | **4** | **Strongly**  **agree  5** |
| --- | --- | --- | --- | --- |
|  |  |  |  |  |

1. I felt very confident using the system.

| **Strongly**  **disagree**  **1** | **2** | **3** | **4** | **Strongly**  **agree  5** |
| --- | --- | --- | --- | --- |
|  |  |  |  |  |

1. I needed to learn a lot of things before I could get going with this system

| **Strongly**  **disagree**  **1** | **2** | **3** | **4** | **Strongly**  **agree  5** |
| --- | --- | --- | --- | --- |
|  |  |  |  |  |
