# Supplementary material for: Mixed reality for teaching catheter placement to medical students: a randomized single-blinded, prospective trial
Source: BMC Med Educ. 2020 Dec 16;20:510. doi: 10.1186/s12909-020-02450-5 (PMC7745503; doi:10.1186/s12909-020-02450-5)
Supplement: Supplementary file 4 — Additional file 4. OSCE checklist applied in this study in German language (original version) and English version. [file 12909_2020_2450_MOESM4_ESM.doc]

**1. Theoretische Erklärung der Patientenvorbereitung**

| **Faktum** | **Gesagt (=1 Punkt)** |
| --- | --- |
| Privatsphäre beachten |  |
| Mann: Rückenlagerung, Beine gestreckt |  |
| Frau: Beine angewinkelt und gespreizt (Fersen stehen zusammen) |  |
| Genital sollte vorher gewaschen sein |  |
| Schutzunterlage unter Gesäß |  |

**2. Material/Vorbereitung**

| **Gegenstand** | **Vorhanden (=1 Punkt)** |
| --- | --- |
| Desinfektionsmittel |  |
| Betäubendes Gleitgel |  |
| Katheter |  |
| Urinbeutel |  |
| Füllmedium |  |
| Spritze |  |
| 2 Paar Sterile Handschuhe |  |

**3 .Vorgehensweise**

| **Aktion** | **Vorhanden (=1 Punkt)** |
| --- | --- |
| Katheterset richtig geöffnet |  |
| Desinfektionsmittel eingegeben |  |
| Alle Materialien steril vorbereitet, inklusive Katheter |  |
| Sterile Handschuhe (2 Paar) richtig angezogen |  |
| Desinfektion durchgeführt |  |
| Handschuhe (1. Paar) ausgezogen |  |
| Gleitgel aufgetragen |  |
| Penisstreckung und dann Absenkung bei Anlage |  |
| Urinbeutel steril verbunden |  |
| Blockung korrekt erfolgt |  |
| Katheter zurückgezogen |  |
| Vorhaut zurückgestreift (mündliche Ansage) |  |

**1. Theoretical explanation of patient preparation**

| **Fact** | **Check (=1 Punkt)** |
| --- | --- |
| Respect privacy |  |
| Male positioning: on backside, legs extended |  |
| Female positioning: legs spread, knees bend, heels pointing towards another |  |
| Genital area washed prior to catheter placement |  |
| Protection pad placed under the patient |  |

2. Material/Vorbereitung

| **Item** | **Check (=1 Punkt)** |
| --- | --- |
| Desinfectant |  |
| Numbing lubricant |  |
| catheter |  |
| Urine reservoir |  |
| Blocking syringe |  |
| syringe |  |
| 2 pairs of sterile gloves |  |

**3 .Vorgehensweise**

| **Action** | **Check (=1 Punkt)** |
| --- | --- |
| Correct opening of catheter set |  |
| Desinfectant filled in container |  |
| All material (incl. catheter) prepared sterile |  |
| Put on sterile gloves correctly (2 pairs) |  |
| Performed desinfection |  |
| Took off one pair of gloves |  |
| Applied lubricant |  |
| Penis stretching and lowering during placement |  |
| Sterile connection of urine resoervoir |  |
| Blocking of catheter correctly performed |  |
| Catheter pulled back after blocking |  |
| Replacement of foreskin (oral explanation, not available on dummy) |  |
