# Supplementary material for: Mixed reality for teaching catheter placement to medical students: a randomized single-blinded, prospective trial
Source: BMC Med Educ. 2020 Dec 16;20:510. doi: 10.1186/s12909-020-02450-5 (PMC7745503; doi:10.1186/s12909-020-02450-5)
Supplement: Supplementary file 5 — Additional file 5. General steps for bladder catheter placement as thaught in this study. [file 12909_2020_2450_MOESM5_ESM.docx]

General steps for bladder catheter placement taken in this study

1. Sterile preparation of required material including disinfection fluid

2. Correct dressing using 2 sets of sterile gloves

3. Disinfection and sterile dressing of patient including urethral disinfection

4. Removal of 1 pair of sterile gloves

5. Lubrification of urethra.

6. Urethral intubation with Foley catheter and correct method of advancing catheter.

7. Filling the catheter balloon.

8. Connection with urinary drainage reservoir.

9. Replacement of foreskin/foreskin pulled back down.
